# Neuroprotective Effect of Chlorogenic Acid on Mitochondrial Dysfunction-Mediated Apoptotic Death of DA Neurons in a Parkinsonian Mouse Model

**DOI:** 10.1155/2020/6571484

**Published:** 2020-05-27

**Authors:** Saumitra Sen Singh, Sachchida Nand Rai, Hareram Birla, Walia Zahra, Aaina Singh Rathore, Hagera Dilnashin, Richa Singh, Surya Pratap Singh

**Affiliations:** ^1^Department of Biochemistry, Institute of Science, Banaras Hindu University, Varanasi 221005, India; ^2^Centre of Biotechnology, University of Allahabad, Prayagraj 211002, India

## Abstract

Mitochondrial dysfunction and oxidative stress characterize major factors involved in the activation of complex processes corresponding to apoptosis-mediated neuronal senescence of dopaminergic neurons (DA) in Parkinson's disease (PD). Here, we evaluated the molecular mechanisms participating in the treatment of a 1-methyl-4-phenyl-1,2,3,6-tetrahydopyridine- (MPTP-) intoxicated PD mouse model in response to chlorogenic acid (CGA). The results indicate that CGA treatment significantly improved the motor coordination of the MPTP-intoxicated mice. CGA also alleviated the fall in activity of mitochondrial complexes I, IV, and V in accordance with ameliorating the level of superoxide dismutase and mitochondrial glutathione in the midbrain of MPTP-induced mice. CGA inhibited the activation of proapoptotic proteins including Bax and caspase-3, while elevating the expression of antiapoptotic protein like Bcl-2 consequently preventing the MPTP-mediated apoptotic cascade. The study also revealed the improved phosphorylation state of Akt, ERK1/2, and GSK3*β* which was downregulated as an effect of MPTP toxicity. Our findings signify that CGA may possess pharmacological properties and contribute to neuroprotection against MPTP induced toxicity in a PD mouse model associated with phosphorylation of GSK3*β* via activating Akt/ERK signalling in the mitochondrial intrinsic apoptotic pathway. Thus, CGA treatment may arise as a potential therapeutic candidate for mitochondrial-mediated apoptotic senescence of DA neurons in PD.

## 1. Introduction

Research has made it quite prominent that aging plays a prime factor in the onset and progression of the sporadic form of the neurodegenerative disorder Parkinson's disease (PD) characterized by the progressive depletion in the dopaminergic (DA) neurons along with the formation and accumulation of Lewy bodies primarily in the substantia nigra pars compacta (SNpc) region of the midbrain and striatum [1, 2]. While the exact mechanism of PD is yet to be elucidated, promising evidences indicate that oxidative stress and mitochondrial dysfunction play a crucial part in its pathogenesis [3–5]. Lewy body formation, comprising aggregates of abnormal or misfolded *α*-synuclein protein, is a hallmark feature of both familial and sporadic forms of PD. *α*-Synuclein undergoes aggregation predominantly in the cytoplasm of the neurons present in SN and leads to multifactorial etiopathogenesis of PD [6, 7]. Numerous pathogenic signals including increased oxidative stress, mitochondrial impairment, ubiquitin-proteasomal dysfunction, and activation of apoptotic cascade along with progressive loss of DA neurons cause the pathogenesis of PD [8, 9]. The clinical evidences from PD patients show the formation of free radicals consisting of reactive oxygen species (ROS), reactive nitrogen species (RNS), mitochondrial dysfunction, adenosine triphosphate (ATP) depletion, and the initiation of caspase-mediated apoptotic cascade in the SN [10].

At the present time, the chief treatment options for PD are dopamine agonists or L-DOPA which simply provide symptomatic relief and show less efficiency with prolonged use resulting in disabling fluctuations and dyskinesias [11]. Therefore, finding treatments with neuroprotective potential against PD in correspondence with effectiveness in relieving symptoms is the main therapeutic target for slowing disease progression. Emerging evidences prove the vital role of apoptosis in PD pathogenesis. Hence, the apoptotic pathway can be prohibited by providing neuroprotection through the use of phytochemicals [12, 13].

Various cell culture models and animal models of PD have been developed using environmental neurotoxins including rotenone, 6-hydroxydopamine, and 1-methyl-4-phenyl-1,2,3,6-tetrahydropyridine (MPTP) [14]. The charged metabolite of MPTP is 1-methyl-4-phenylpyridinium (MPP^+^) acting as the main toxic agent against the DA neurons in the brain of MPTP-intoxicated *in vivo* models [15, 16]. MPP^+^ readily enters the nigrostriatal neurons via dopamine transporters and affects the SN neuronal loss contributing to striatal dopamine depletion further causing a parkinsonian syndrome. It also disturbs the mitochondrial membrane potential causing mitochondrial stress. It is reported to be an inhibitor of complex I of the electron transport chain (ETC) and contributes to considerable oxidative stress along with ATP depletion, consequently leading to neuronal loss [17]. MPP^+^ activates the proapoptotic proteins, making the mitochondrial membrane permeable for cytochrome c to be released into the cytosol. Associated signalling inactivates protein kinases (MAPKs) which might have a neuroprotective role.

Akt and ERK signalling pathways play crucial parts in neuroprotection via cell differentiation, proliferation, survival, and apoptosis [18, 19]. Numerous recent researches have highlighted the neuroprotective role of plant-based compounds and polyphenols in PD against neurotoxins and neuroinflammation, by promoting cell survival through their antioxidative, anti-inflammatory, and immunomodulatory effects [20–22]. Evidences prove the pivotal role of polyphenols in replenishing the neurons via increased activity of ETC, regulating the effects on redox potential, and restraining the apoptosis as a result of increased mitochondrial biogenesis [23–25]. CGA, a type of phenolic acid, is reported to demonstrate antiapoptotic, anti-inflammatory, antioxidative, neurotrophic, anticancerous, and neuroprotective properties. CGA is formed as an ester of the cinnamic acids including caffeic acid and quinic acid forming the second major component of coffee after caffeine [26–28]. In our previous study, we reported the antioxidative and anti-inflammatory property of CGA against the neurotoxic effect of MPTP in mice [26]. The present study was undertaken to assess the neuroprotective effect of CGA in an MPTP-induced PD mouse model via modulation of Akt, ERK1/2, and GSK3*β* signalling pathways. Under this context, the effect of CGA was evaluated against MPP^+^-mediated DA neuronal injury in MPTP-intoxicated mice showing its neuroprotective role via the activation of Akt and ERK1/2 signalling pathways by inhibiting the mitochondrial dysfunction. Our findings may be helpful in designing a CGA-based treatment strategy against PD.

## 2. Chemical and Reagents

### 2.1. Reagents and Antibodies

Mannitol, sodium dihydrogen phosphate, bovine serum albumin (BSA), oxidized cytochrome c, digitonin, disodium hydrogen phosphate, potassium chloride, ammonium chloride, and reduced nicotinamide adenine dinucleotide phosphate (NADPH) were purchased from Sisco Research Laboratories (SRL, Mumbai, India). MPTP, DABCO, 5,5-dithiobis 2-nitrobenzoic acid (DTNB), normal goat serum (NGS), and chlorogenic acid were obtained from Sigma-Aldrich (St. Louis, MO, United States). Sucrose and sodium carbonate were purchased from Merck (Darmstadt, Germany), and EGTA and sodium dodecyl sulphate (SDS) were obtained from HiMedia (Mumbai, India). Glycylglycine buffer, sodium fluoride, paraformaldehyde, Tris buffer, and trichloroacetic acid were bought from LobaChemie, India. Primary antibodies for Akt, p-Akt, caspase-3, ERK, p-ERK, and Bcl-2 were acquired from Abcam Life Science, Biogenuix Medsystems Pvt. Ltd. (New Delhi, India), and primary antibodies for GSK3*β*, p-GSK3*β*, TH, *β*-actin, and Bax were purchased from Santa Cruz Biotechnology (Santa Cruz, CA, United States).

### 2.2. Experimental Animals

The experiment was conducted using male Swiss albino mice (25 ± 5 g), obtained from the Institute of Medical Sciences' animal facility, Banaras Hindu University, Varanasi (India). The mice were kept in clean polypropylene cages with constant light-dark cycles of 12 h prior to the start of experiment. Mice were provided with *ad libitum* water and standard diet pellet until the dosing was completed. The experimental protocol was established according to the Animal Ethics Committee's guidelines of Banaras Hindu University, Varanasi, India.

#### 2.2.1. Animal Dosing

Experimental animals were randomized into four groups: control, MPTP, MPTP+CGA, and CGA (*n* = 10/group). The dosing was administered in accordance with our previous study with some modifications [26]. The mice in the control group received normal saline orally, once consequently for 24 days. In MPTP+CGA and CGA groups, the oral administration of 50 mg/kg of CGA was done, once daily for 24 days. Except for the control and drug-only groups, all the mice received MPTP (30 mg/kg) intraperitoneally, dissolved in normal saline for five consecutive days (from the 20^th^ to 24^th^ day of CGA dosing). After completion of dosing, a behavioral test was performed from the 25^th^ to 28^th^ day, and thereafter, mice were sacrificed to isolate the brains for the mitochondrial dysfunction assay, Western blotting, and immunohistochemical analysis.

#### 2.2.2. Behavioral Test

To assess the effect of MPTP intoxication on motor function impairment in the parkinsonian mouse model, four behavioral tests were conducted, including the rotarod test, pole test, catalepsy test, and traction test.

#### 2.2.3. Rotarod Test

The training for 3 consecutive days was given to the animals from all the groups at a speed of 5 rpm for the rotarod test. Thereafter, the time on the rotarod for each animal was noted down for maximum 5 min. The test was done four times, and finally, the average time for which the mice stayed on the rotarod was noted down [29]. After the completion of dosing, the rotarod test was again conducted and the time spent on the instrument was noted down for each mouse.

#### 2.2.4. Pole Test

Bradykinesia in PD mouse models is usually evaluated by this test [30]. The test was done after the last MPTP injection was given. Mice were supported on the top of a pole (diameter 10 mm, height 52 cm, with a rough surface). The T-turn (time to turn) was recorded accordingly to the time taken by mice to step down the length of the pole.

#### 2.2.5. Traction Test

Equilibrium and the muscle strength are measured by performing the traction test [30], and it was also conducted on the same day as the pole test. The forepaws of a mouse were located on a suspended horizontal bar, while its hind limb placements were scored from 1 to 3, with the lowest score indicating the severe motor and balance impairment. The following criteria were used to evaluate the score: the score was 3, if both hind limbs seized the bar, while it was 2 or 1, if one or no hind limb seized the bar, respectively.

#### 2.2.6. Catalepsy Test

Catalepsy is a state of the behavioral test that characterizes the muscle stiffness in which the mice fail to change its posture which is imposed to them and is estimated by making the animals to stand on a wooden platform where their hind limbs were placed on a wood and forelimbs on the ground [31]. The intensity of catalepsy was measured when a mouse moved its hind limbs from a wooden platform (3 cm). The mice were adapted for 3 min, and if the latency exceeds more than 180 sec, the test was ended.

### 2.3. Mitochondrial Parameter Analysis

#### 2.3.1. Isolation of Mitochondria

Differential centrifugation was done to isolate the mitochondrial pellet from the mouse brain [32]. The midbrain regions of the mice were homogenized in homogenizing buffer, prepared by mixing 5 mM HEPES, 225 mM mannitol, 1 mM ethylene glycol tetra acetic acid (EGTA), 75 mM sucrose, and 1 mg/ml BSA (pH 7.4). Centrifugation of the homogenates was done at 2,000 g for 30 min at 4°C. The pellet was disposed, and the supernatant obtained was again set to centrifugation for 10 min at 12,000 g. The resultant pellet constituting the mixture of mitochondria and synaptosomes was dissolved in the homogenizing buffer containing digitonin (0.02%). Crude mitochondrial fraction was finally obtained by the centrifugation of the suspended mixture for 10 min at 12,000 g. Homogenizing buffer without BSA and EGTA was used to wash the crude mitochondrial fraction twice, and finally, the resuspension was done in phosphate buffer (50 mM, pH 7.4). The Bradford assay was performed to estimate proteins in all the samples. The assays were done within 24 h of the mitochondrial isolation using 20 *μ*g proteins.

#### 2.3.2. Complex I-III Assay

The assay of complexes I-III was done by incorporating the method of Sood et al. in our study [33]. In this assay, the catalytic oxidation of NADH to NAD^+^ is done by subsequently reducing cytochrome c. The reaction mixture was prepared by the addition of glycylglycine buffer (0.2 M, pH 8.5), NADH (6 mM in 2 mM glycylglycine buffer), and cytochrome c (10.5 mM). 20 *μ*g mitochondrial protein was added to the reaction mixture, and the absorbance change was recorded at 550 nm for 2 min. At 340 nm, the extinction coefficient for NADH is 6.22/mM/cm. The activity of the enzyme was expressed in terms of nmol NADH oxidized/min/mg protein.

#### 2.3.3. Complex IV Assay

Complex IV activity was determined by measuring the oxidation of reduced cytochrome c by complex IV at 550 nm [33]. The reaction mixture consisted of mitochondrial protein (20 *μ*g) along with 10 mM phosphate buffer (pH 7.4), reduced cytochrome c, and potassium ferricyanide. Cytochrome c (oxidized) solution (10 mg/ml) was used to prepare cytochrome c (reduced), by adding few crystals of sodium borohydride in it. Absorbance change was monitored for about 3 min. The activity of complex IV was calculated in nmol cytochrome c oxidized/min/mg of protein.

#### 2.3.4. Complex V Assay

Griffiths and Houghton's method was used to perform the mitochondrial ATPase test [34]. In this assay, the amount of inorganic phosphorus liberated is measured that is obtained by the hydrolysis of ATP to ADP. The incubation of the reaction mixture (1.0 ml) containing the mitochondrial sample and ATPase buffer (2 mM MgCl_2_, 5 mM ATP, and 50 mM Tris HCl, pH 8.5) was done for 5 min at 30°C. After the addition of 10% TCA, the reaction mixture was spun at 3,000 g for 10 min. Phosphorus was assayed, and the results were expressed as nmol inorganic phosphate (Pi) liberated/min/mg protein.

#### 2.3.5. Mitochondrial GSH

Mitochondrial GSH (reduced) content was estimated by using the mitochondrial protein sample (20 *μ*g) suspended in phosphate buffer. The addition of 25% trichloroacetic acid (TCA) was done to the samples, followed by a brief centrifugation at 1,500 g. The supernatant was collected, and 5,5-dithiobis 2-nitrobenzoic acid (DTNB) was added that reacts with thiol groups to form 2-nitro-5mercapto benzoic acid. Absorbance was recorded at 412 nm. Further, commercially available GSH was used to prepare the standard curve, and the amount was expressed as mmol of GSH/g mitochondrial protein [35].

#### 2.3.6. Manganese SOD

The activity of manganese superoxide dismutase (Mn-SOD) was estimated by adding hydrogen peroxide that selectively inhibits Cu/Zn-SOD, in order to distinguish the enzyme's activity from that of Cu/Zn-SOD. The reaction mixture consists of sodium carbonate (50 mM), EDTA (0.1 mM), Triton-X (0.6%), and NBT (90 mM) and hydroxylamine hydrochloride [35]. Hydroxylamine hydrochloride undergoes photooxidation to produce superoxide that further reduces nitroblue tetrazolium (NBT) in the reaction medium, and this reaction was inhibited by SOD. The reading was taken at 560 nm for 3 min, and the SOD activity was expressed as units. The one enzymatic unit of SOD was defined as the amount of enzyme required for 50% inhibition.

### 2.4. Western Blot (WB) Analysis

The SN of the mouse brain was homogenized using the lysis buffer (RIPA) and agitated for 2 h at 4°C. The homogenate was centrifuged (12,000 rpm, 30 min) to collect the supernatant, and the Bradford assay was performed to quantify the protein concentration. The total protein extract (50 *μ*g) was loaded onto the polyacrylamide gels. After electrophoresis, proteins were transblotted to PVDF membranes and sequentially incubated overnight at 4°C with Bax (1 : 1000), Bcl-2 (1 : 800), caspase-3 (1 : 1000), p-Akt (1 : 1000), Akt (1 : 1000), p-ERK1/2 (1 : 1000), ERK1/2 (1 : 800), p-GSK3*β* (1 : 1000), GSK3*β* (1 : 1000), and *β*-actin (1 : 1000) and then with a horseradish peroxidase- (HRP-) conjugated secondary antibody for 2 h at room temperature. TBST washing (twice) separated each step. Blots were visualized using the DAB system (buffer+substrate+chromogen), and for each band, the relative density was calculated with respect to that of *β*-actin. Quantity One software (Windows, Bio-Rad) was used to indicate the expression of relative density.

### 2.5. Immunohistochemistry (IHC)

Mice from each group were sacrificed using pentobarbital perfused intracardially with chilled 0.9% saline solution and further using 4% paraformaldehyde solution (prepared in 0.1 M phosphate-buffered saline, pH 7.4). Again, paraformaldehyde (10%) was used to postfix the brains overnight, and further, 30% sucrose solution was used to immerse the brains. Using the standard protocol, immunohistochemical staining was performed for TH, p-Akt, p-Erk1/2, and p-GSK3*β* [36]. Coronal brain sections were cut to 20 *μ*m thickness using a cryotome (Leica, Wetzlar, Germany). Tissue sections were washed with 0.01 M PBS (pH 7.4) 2 × 10 min, followed by 1 h blocking with 10% NGS in PBST and then 1% BSA-PBST. The sections were rinsed with PBS and incubated with primary antibodies for TH (1 : 1000), p-Akt1 (1 : 500), p-ERK1/2 (1 : 500), and p-GSK3*β* (1 : 500) at 4°C (16 h). The brain sections were then treated with FITC-conjugated (for anti-mice primary) and TRITC-conjugated (for anti-rabbit primary) secondary antibodies (diluted in 1% BSA-PBS) at room temperature (2 h). Tissue sections were washed thrice at each step with 1% BSA-PBS and PBS, respectively, and mounted with polyvinyl alcohol with DABCO. Images were captured with a fluorescent microscope (Nikon, Thermo Fisher Scientific). All images were then processed by ImageJ software (NIH, United States). Results expressed as a % area were reported.

### 2.6. Statistical Analysis

The analysis of data was done by one-way analysis of variance (ANOVA) using the Student–Newman–Keuls test and by Student's two-tailed *t*-test using GraphPad Prism software. The results are expressed as the mean ± SEM. *p* values < 0.05 were considered statistically significant.

## 3. Results

### 3.1. Behavioral Parameter Analysis

**Figure 1:**
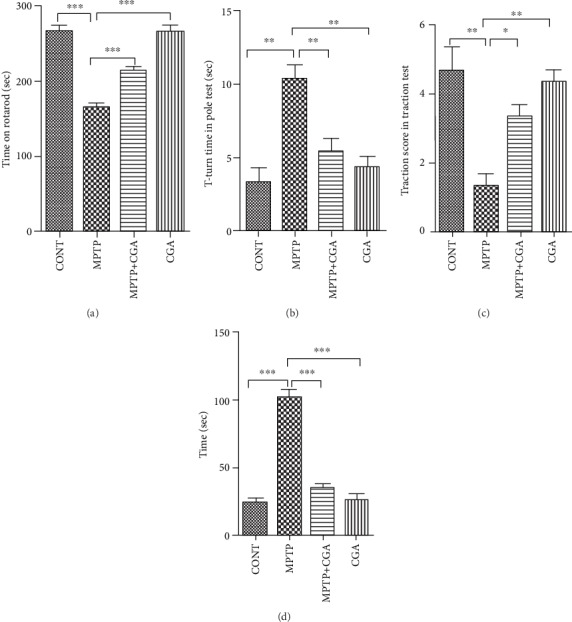
Mouse behavioral analysis. CGA mediated alteration in the neurobehavior of MPTP-injected mice. (a) Time on the rotarod test. (b) T-turn time in the pole test. (c) Traction score in the traction test. (d) Cataleptic test. Values are represented in the form of mean ± SEM (*n* = 10). ^∗^*p* < 0.05, ^∗∗^*p* < 0.01, and ^∗∗∗^*p* < 0.001. Results were studied using the one-way ANOVA and further using the Newman-Keuls test.

The results from our study suggested that the MPTP-intoxicated animal falls early from the rotarod in comparison to the control (*p* < 0.001, Figure 1(a)). On the other hand, when CGA was administered to the MPTP-treated mice, the time on the rotarod was significantly increased (*p* < 0.001). The pole test clued that MPTP-treated mice showed prolonged locomotor activity time (*p* < 0.01Figure 1(b)). The time duration was shortened significantly (*p* < 0.01) in pretreated mice with CGA, suggesting that bradykinesia induced by MPTP was alleviated by CGA treatment. Results of the traction test (Figure 1(c)) showed that PD mice have decreased strength and latency time as their hind limb grip score was lower (*p* < 0.01) than that of the control group. However, pretreatment with CGA increased the traction score (*p* < 0.05). This indicates that prophylactic treatment with CGA could mitigate the initial lesions induced by MPTP. Catalepsy was observed among the MPTP group of mice after MPTP injection. Latency in the catalepsy test among the MPTP group showed increased tendency with time (*p* < 0.001, Figure 1(d)), and the observed data was of considerable difference from that of the control group, while the mice treated with CGA showed little latency time (*p* < 0.001) than the MPTP-intoxicated group. Any significant changes did not appear in the neurobehavioral activity between the control and CGA-alone-treated animals. Thus, results have suggested that CGA improved the neurobehavioral lesion of MPTP-injected PD mice.

### 3.2. CGA Modulates MPTP-Induced Mitochondrial ETS Impairment in Mice

**Figure 2:**
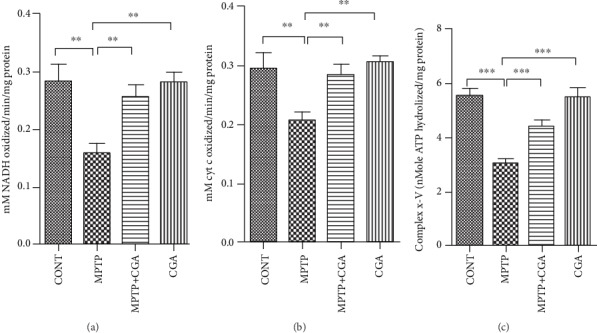
CGA mediated alterations on mitochondrial complexes I-III (a), IV (b), and V (c) of the electron transport chain in the midbrain of mice. Values are represented in the form of mean ± SEM (*n* = 5). ^∗^*p* < 0.05, ^∗∗^*p* < 0.01, and ^∗∗∗^*p* < 0.001. Results were studied using the one-way ANOVA and further using the Newman-Keuls test.

Oxidative phosphorylation in mitochondria produces ATP as energy, and this process requires the organized action of five enzyme complexes located in the inner membrane. Catastrophic consequences can be observed by any defect in these energy-generating complexes, not only because of the ATP loss but also due to the downstream functional loss. Therefore, the consequences of MPTP intoxication on the energy metabolism in the mouse brain were studied. MPTP being a known complex I inhibitor of the electron transport chain (ETC) was analysed to study its effect on the activities of various complexes in the respiratory chain. As Figure 2 depicted, the effect of CGA treatment on MPTP induced alterations in the complex I-III (Figure 2(a)), IV (Figure 2(b)), and V activities (Figure 2(c)). There was significant attenuation in the activities of complexes I-III (*p* < 0.01), complex IV (*p* < 0.01), and complex V (*p* < 0.001) in MPTP-intoxicated PD mice as compared to control mice. CGA administration before MPTP intoxication to the animals helped improve the activities of complexes I-III (*p* < 0.01), complex IV (*p* < 0.01), and complex V (*p* < 0.001) in comparison with MPTP-intoxicated mice. No potential change was observed when the control group was treated with CGA.

### 3.3. Mitochondrial Glutathione and Superoxide Dismutase Level Analysis

**Figure 3:**
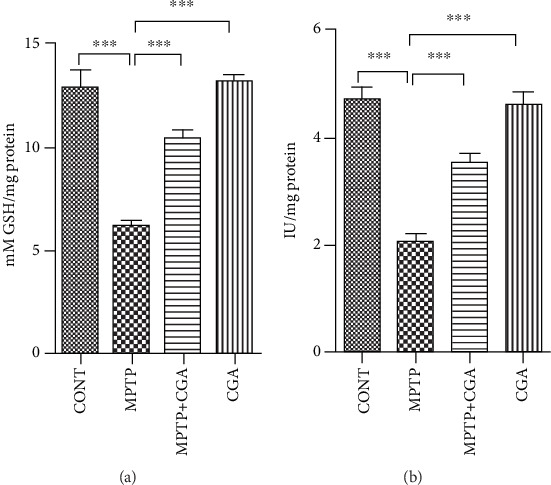
Alterations in mitochondrial antioxidant defence in respective treatment groups. (a) Mitochondrial reduced glutathione level. (b) Mitochondrial superoxide dismutase level. Values are represented in the form of mean ± SEM (*n* = 5). ^∗^*p* < 0.05, ^∗∗^*p* < 0.01, and ^∗∗∗^*p* < 0.001. Results were studied using the one-way ANOVA and further using the Newman-Keuls test.

Further, the increase in oxidative stress resulting in the reduced function of ETC leads to the utilization of mitochondrial GSH (mtGSH, reduced) and reduction in activity of Mn-SOD (Figures 3(a) and 3(b)). In the MPTP-treated group, a significant reduction (*p* < 0.001) in the levels of mtGSH was observed when compared to the control group. Consequently, a decline in the Mn-SOD activity (*p* < 0.001) was also analysed with MPTP intoxication (Figure 2(b)). On the other hand, CGA has reduced the ROS generation and ameliorated the function of ETS which has ultimately increased the level of mtGSH (*p* < 0.001) and Mn-SOD (*p* < 0.001) significantly.

### 3.4. CGA Inhibited the MPTP-Induced Activation of Mitochondrial Apoptosis Signalling in SN of Mice

**Figure 4:**
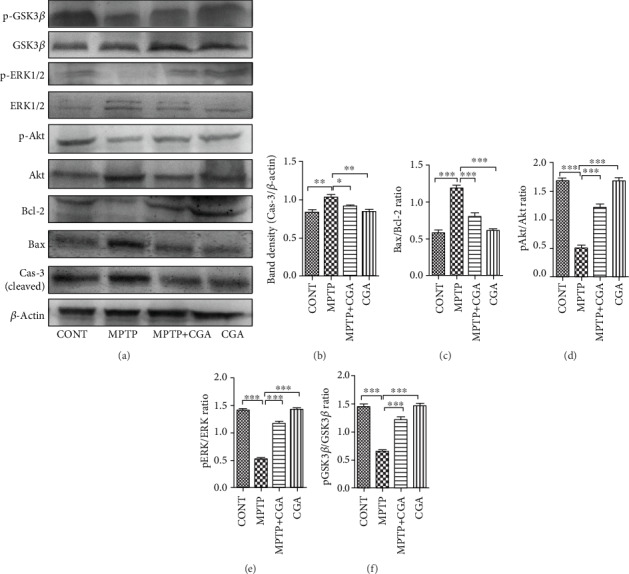
(a–f) Relative expression of Bax, Cas-3, Bcl-2, p-Akt, p-ERK1/2, and p-GSK3*β* in SN of mice was studied using the Western blot technique and densitometry analysis of proteins. CGA suppressed MPTP-induced apoptosis via Akt, GSK3*β*, and ERK1/2 signalling pathways in parkinsonian mice. It has normalized the deregulated expression of Bax, Bcl-2, and Cas-3 in MPTP-injected mice (a, b). *β*-Actin protein served as an internal control. Values are represented in the form of mean ± SEM (*n* = 5). ^∗^*p* < 0.05, ^∗∗^*p* < 0.01, and ^∗∗∗^*p* < 0.001. Results were studied using the one-way ANOVA and further using the Newman-Keuls test. Abbreviations: CGA: chlorogenic acid; MPTP: 1-methyl-4-phenyl-1,2,3,6-tetrahydropyridine; SEM: standard error of the mean.

Various apoptosis-linked molecules were investigated to study the effect of CGA on MPTP-induced cell apoptosis rate (Figures 4(a)–4(c)). The MPTP-intoxicated group showed a considerable increment in the Bax/Bcl-2 ratio (*p* < 0.001) as compared to the control group whereas in CGA-treated PD mice, the Bax/Bcl-2 ratio (*p* < 0.001) was observed to be significantly reduced, which shows the antiapoptotic property of CGA in the parkinsonian mouse model (Figure 4(b)). The expression level of cleaved caspase-3 (*p* < 0.01) was observed to be elevated in the SN of PD mice whereas attenuated expression of cleaved caspase-3 (*p* < 0.05) was observed in the drug-treated group, which shows the antiapoptotic property of CGA in the parkinsonian mouse model (Figure 4(c)).

### 3.5. CGA Ameliorates the Dysregulation of Akt and ERK Activation and GSK3*β* Phosphorylation in the MPTP-Induced Parkinsonian Mouse Model

**Figure 5:**
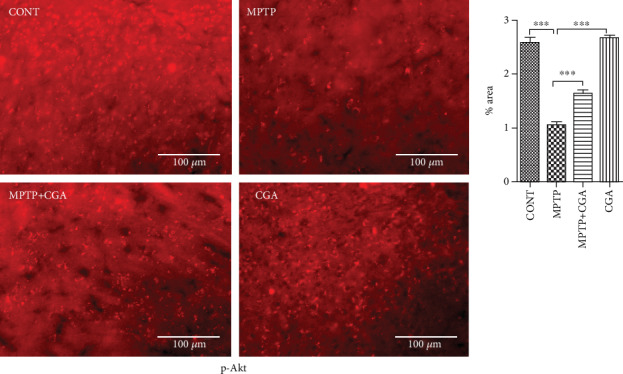
Immunohistochemical staining to analyse the expression of p-Akt in SN of different experimental groups. CGA administration enhanced the expression of p-Akt in SN of parkinsonian mice (20x magnification after staining). Values are expressed as mean ± SEM (*n* = 5). ^∗^*p* < 0.05, ^∗∗^*p* < 0.01, and ^∗∗∗^*p* < 0.001. Results were studied using the one-way ANOVA and further using the Newman-Keuls test.

**Figure 6:**
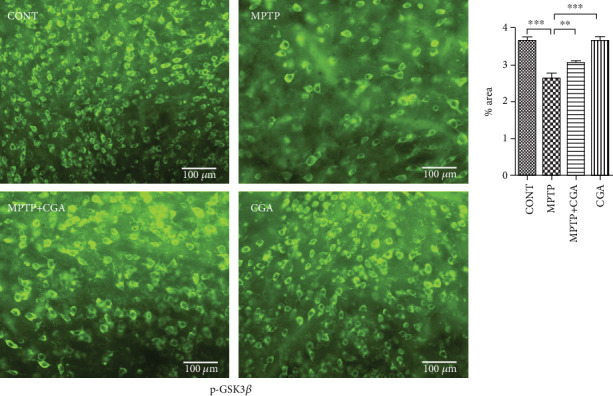
Immunohistochemical staining to analyse the expression of p-GSK3*β* in SN of different experimental groups (a, b). Profound expression of p-GSK3*β* in the CGA-administered group compared to the MPTP-intoxicated PD mice (20x). Values are represented as mean ± SEM (*n* = 5). ^∗^*p* < 0.05, ^∗∗^*p* < 0.01, and ^∗∗∗^*p* < 0.001. Results were studied using the one-way ANOVA and further by the Newman-Keuls test.

**Figure 7:**
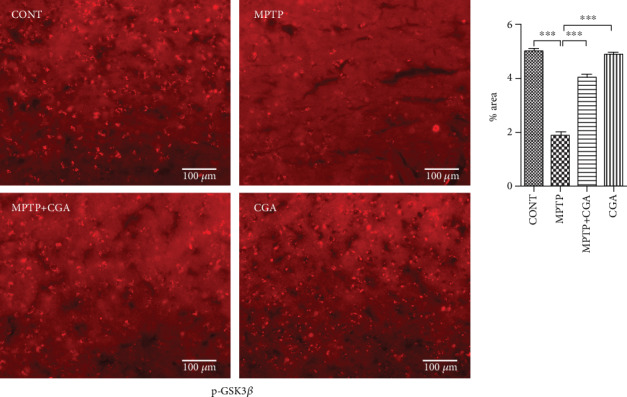
Immunohistochemical staining to analyse the expression of p-ERK1/2 in SN of different experimental groups. Upregulated expression of p-ERK1/2 due to CGA administration in parkinsonian mice (20x). Values are represented as mean ± SEM (*n* = 5). ^∗^*p* < 0.05, ^∗∗^*p* < 0.01, and ^∗∗∗^*p* < 0.001. Results were studied using the one-way ANOVA and further by the Newman-Keuls test.

Expression of Akt, ERK1/2, and GSK3*β* was examined by WB (Figures 4(a) and 4(d)–4(f)) and immunohistochemical analysis techniques (Figures 5–7). Akt and ERK1/2 are the major downstream key components of the MAPK signalling pathway. Our WB data emphasised that MPTP treatment significantly attenuated the p-Akt/Akt ratio (*p* < 0.001) compared with the control group, whereas in CGA treatment, the p-Akt/Akt ratio (*p* < 0.01) was observed to be elevated in PD mice (Figure 4(d)). Similar results were also observed through the IHC technique showing reduced Akt phosphorylation in MPTP-treated mice (*p* < 0.001) whereas increased p-Akt was observed in the case of CGA-treated PD mice (Figure 5). Furthermore, WB results depicted downregulation in the p-ERK1/2/ERK1/2 ratio in MPTP-treated mice (*p* < 0.001). On the contrary, the p-ERK1/2/ERK1/2 ratio (*p* < 0.001) was upregulated in CGA-treated PD mice (Figure 4). Likewise, in IHC, the ERK1/2 phosphorylation was seen to be decreased on MPTP intoxication (*p* < 0.001) whereas CGA treatment has significantly increased the phosphorylation of ERK1/2 (*p* < 0.001) (Figure 7).

GSK3*β* has recently been linked to the mitochondrial intrinsic apoptosis. The WB result for GSK3*β* revealed a reduced p-GSK3*β*/GSK3*β* ratio (*p* < 0.001) in MPTP-intoxicated mice whereas in CGA-treated PD mice, the ratio of p-GSK3*β*/GSK3*β* was seen to be increased (*p* < 0.001, Figure 4(f)). To further confirm the results, IHC was performed presenting a substantial decrease in GSK3*β* phosphorylation after MPTP treatment in comparison with the control group (*p* < 0.01). On the other hand, CGA administration leads to the enhanced phosphorylation of GSK3*β* when compared to that of MPTP-intoxicated mice (*p* < 0.001, Figure 6).

### 3.6. CGA Ameliorates MPTP-Induced Degeneration of TH-Positive DA Neurons in SN of PD Mice

**Figure 8:**
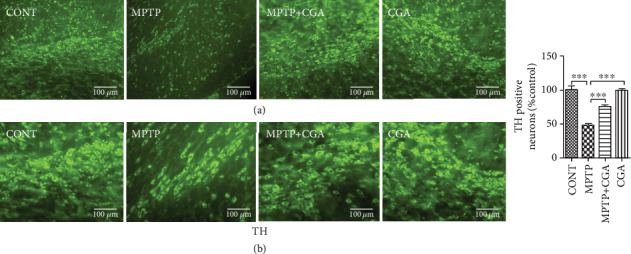
(a, b) Immunohistochemical staining to analyse the expression of TH-positive DA neurons in SN of different experimental groups. CGA protected the TH-positive DA neurons in MPTP-induced mice. (a) 10x and (b) 20x. TH immunoreactivity in SN of mice. Values are represented as mean ± SEM (*n* = 5). ^∗^*p* < 0.05, ^∗∗^*p* < 0.01, and ^∗∗∗^*p* < 0.001. Results were studied using the one-way ANOVA and further by the Newman-Keuls test.

IHC of TH was performed to assess the loss of DA neurons in SN of MPTP-intoxicated mice (Figure 8). The data obtained in this study reveals the significant reduction of DA neurons in SN of an MPTP-intoxicated mouse as compared to control (*p* < 0.001). Alternatively, CGA pretreatment has done a significant increment in the expression of TH (*p* < 0.01) in SNpc of an MPTP-intoxicated mouse as compared with the MPTP-induced PD mouse model. Therefore, CGA administration was beneficial in protecting the DA neurons from MPTP-induced toxicity, as suggested by our findings.

## 4. Discussion

PD occurs due to multiple factors, and various pathways interact to induce the neurotoxic pathways that result in the loss of DA neurons in SN [6, 9]. The pathways that lead to neurodegeneration are influenced by each other, yet their contribution is independent of each other. All the involved processes work in an interdependent manner influencing one another and finally leading to the neuronal senescence [37, 38]. Hence, neuroprotection in the case of PD can only be achieved when combination of drugs or treatments targeting the collective pathways is used for PD therapy. In this context, it has been seen that CGA shows multiple biological effects such as antioxidant, anti-inflammatory, neuroprotective, and neurotrophic activities [27, 39–41]. In this study, CGA act as a potent neuroprotective agent by preventing mitochondrial dysfunction and suppressing the apoptotic death of DA neurons. Also, these neuroprotective effects of CGA were found to be mediated by Akt, ERK1/2, and GSK3*β* pathways. Different behavioral tests such as pole, catalepsy, rotarod, and traction tests have helped determine severe motor abnormalities in MPTP-intoxicated PD animal models [42–45] (Figure 1). Similar coordination deficits and motor impairment were also seen in our study too upon MPTP toxicity in mice, thereby validating our mouse model of PD in accordance with previous researches, whereas the neurobehavioral lesion was found to be alleviated by CGA as suggested by the behavioral tests which is in correspondence with earlier reports [30, 46–48].

The key pathogenic events of PD mainly constitute oxidative stress and mitochondrial dysfunction, and these two further lead to the onset of cell apoptosis [49, 50]. The neurotoxin MPTP serves as an excellent animal model for PD as it mimics the symptoms of PD extensively. The neurotoxin after crossing the blood-brain barrier (BBB) is acted upon by monoamine oxidase B to get converted into MPP^+^ and enters into DA neurons specifically via the dopamine transporter. MPP^+^ is the toxic metabolite of MPTP, and its accumulation inside the neurons leads to the inhibition of complex I of mitochondria and induces the ROS generation and depletion of ATP, prompting to the loss of the DA neurons [51, 52]. Both *in vivo* and *in vitro* models have been studied for the effects of antioxidants on the reduced mitochondrial dysfunction along with the enhanced growth of certain bioactive compounds in the mitochondria [53, 54]. In this study, MPTP intoxication reduced the activity of complexes I-III, complex IV, and complex V of ETS (Figure 2). Thus, the pattern of electron transfer was disturbed by MPTP toxicity, which has led to the reduction in the effectiveness of the enzymes NADH dehydrogenase and cytochrome oxidase causing structural damage to the mitochondria leading to its dysfunction. Further, because of the reduced functioning of ETC, oxidative stress arises triggering the reduced activity of Mn-SOD and excessive consumption of mitochondrial GSH (mtGSH, reduced). A research done by Davey et al. in 1998 proposed that the reduced activity of complex I occurs because of the depletion in the level of GSH in PC12 cells, which also leads to the abolition of the threshold effect [55]. The mechanism involved in the threshold hindrance of complexes I-III by the GSH level is still unknown. The antioxidant GSH is known to protect the mitochondria from lipid peroxidation [56], and hence, its depletion might make complexes I-III prone to the attack of the free radicals. Also, in previous researches, it has been described that the depleted level of GSH causes the degeneration and enlargement of the brain mitochondria [57] and induces the reduced activity of complex IV in the purified brain mitochondrial preparations [58]. The decreased level of the reduced glutathione or the generation of reactive oxygen and nitrogen species by the activated glial cells might be the main factors for the decline in complex IV activity. Moreover, mitochondrial damage also takes place by the production of NO as a result of inflammation-mediated expression of iNOS induction, which causes the loss of the cytochrome oxidase pathway by producing RNS consequently inducing protein nitration in the ETC. Moreover, various *in vivo* studies have reported the pivotal role of GSH in protecting DA neurons against the adverse effects of MPTP on PD [59, 60]. Therefore, the findings suggest that the fall in the reduced GSH level increased the sensitization of mitochondria against the additional metabolic insult resulting in its dysfunction and cell death. However, in our study, CGA was found to be potent in maintaining the appropriate activity of the ETC complexes (Figure 3). Furthermore, as reflected by the enhanced levels of reduced mtGSH and Mn-SOD, CGA was also effective in reducing the burden on the mitochondrial antioxidative defence by inhibiting the generation of the ROS. Thus, the ability of CGA to improve the activities of the complexes of ETC might be due to increased availability of reduced glutathione which is known to be the primary defence of mitochondrial functions.

The mitochondrion, an intracellular powerhouse, plays various crucial roles including energy production, free reactive oxygen and nitrogen species production along with critically modulating the apoptosis, and cell survival which are essential factors of aging. During the pathological alterations, the leakage of cytochrome c and various proapoptotic molecules in the cytoplasm from the mitochondria, as a result of the mitochondrial membrane permeabilization, induces the cascade of events leading to cell death [61]. Mitochondrial dysfunction is generally estimated by measuring the membrane potential which is found impaired in the intrinsic apoptotic pathway [62]. The regulation of caspase activation is maintained as a result of the equilibrium between the expression levels of proapoptotic and prosurvival proteins in the Bcl-2 family [63], although Bcl-2 and Bax have contrasting functions while being from the same Bcl-2 family. While Bcl-2 is known for its antiapoptotic activity, Bax exhibits the proapoptotic function [64]. Numerous studies have suggested that the neurotoxin MPTP disturbs the balance of Bax/Bcl-2, which increases the activity of caspase-3 in the DA neurons [65, 66]. The release of apoptogenic proteins like cytochrome c into the cytosol from mitochondria mediates the activation of caspase-3 which occurs because of the loss of membrane integrity by the action of Bax, hence leading to cell death. Both *in vivo* and *in vitro* studies have suggested the increased expression of caspase-3 upon MPTP toxicity [67]. The activation of caspase-9 provokes the initiation of the apoptotic cascade on the release of cytochrome c into the cytosol after mitochondrial dysfunction. As a result, caspase-3 gets activated by changing the morphology of mitochondria leading to apoptosis [68]. However, CGA treatment has protected the DA neurons from MPTP-induced toxicity and inhibited the apoptosis (Figure 4).

Different cellular processes like growth, proliferation, survival, and apoptosis are regulated by the phosphorylation of some key proteins such as Akt, ERK1/2, and GSK3*β* [69, 70]. The activation of Akt is found to be linked with the survival pathways of various cell types including neuronal cells as reported in several *in vitro* studies. Moreover, the survival of different neurons is linked with different neurotrophic factors, whose activation is mediated by Akt [71–73]. Also, transfection with a constitutively activated form of Akt is observed to promote the survival of the neurons without any other support whereas the cultured neurons were found to be dead when the activation of the kinase was interfered [74]. Studies from different sources have suggested the role of GSK3*β* in the modulation of apoptosis, and the inhibition of GSK3*β* has protected the DA neurons from MPTP toxicity as suggested by different cell and animal models of PD [45, 75, 76]. The localization of GSK3*β* has been mainly observed in the cytosol, but its expression in lower amounts has also been seen in the nucleus and mitochondria [77, 78]. Furthermore, it is also linked with the regulation of the mitochondrial cell death pathway by eliciting numerous stressful conditions within the neurons [78]. Researchers have also suggested that inhibition of GSK3*β* rescues the DA neurons from MPTP toxicity, indicating its association with the pathogenesis of PD [45, 76]. The neurotoxin MPTP has been found to decrease the phosphorylation of Akt and GSK3*β* as suggested by previous studies, leading to the death of the DA neurons [70, 79]. MPTP-induced cell injury is mediated by GSK3*β*, which is the downstream target of Akt [75, 80]. Mitochondrial dysfunction is seen to be facilitated by GSK3*β*, and its inhibition prevents the loss of neurons mediated by the suppression of proapoptotic proteins [76, 81]. In accordance with previous studies, MPTP has decreased the phosphorylation of Akt (Figures 4 and 5) and GSK3*β* (Figures 4 and 6), and CGA on the other hand has protected Akt and GSK3*β* from dephosphorylation in the MPTP-intoxicated mouse model. It has been observed that Akt controls the phosphorylation of GSK3*β* and thereby inhibits its activity [82]. MAPK signalling pathways also play a pivotal role in many cellular events, such as proliferation, differentiation, and apoptosis. The three major MAPK subfamilies have been chiefly characterized including ERK, JNK, and p38 [83]. Among them, ERK is known to enhance the DA neuronal survival rate, as in SH-SY5Y cells, the phosphorylation of ERK was seen to be suppressed after 4 h of MPP^+^ toxicity [84, 85]. Our study also shows the dephosphorylation of ERK upon MPTP intoxication, while the neuroprotective agent CGA was potent in abolishing the neurotoxic effect in the PD mouse model (Figures 4 and 7).

TH immunoreactivity in this study was performed to examine the CGA-mediated alterations on the functions of DA neurons in the SN of the MPTP-intoxicated parkinsonian mouse model [86] (Figure 8). Our immunohistochemical study has shown the reduction expression of TH-positive DA neurons in SN of an MPTP-intoxicated mouse, i.e., the characteristic feature of PD, as suggested by numerous reports [44, 60, 86]. However, CGA has rescued this debilitating effect and hence conferred about protecting the DA neurons against MPTP-induced neurodegeneration in the mouse model of PD.

Hence, our results have therefore suggested that the neuroprotective effect of CGA is mediated by the GSK3*β* phosphorylation-associated Akt/ERK pathway and is potent in inhibiting the mitochondrial intrinsic apoptosis due to MPTP toxicity in the mouse brain.

## 5. Conclusion

This study demonstrates the neuroprotective effect of CGA against MPTP-induced apoptotic cell death in mice. The Bax/Bcl-2 ratio, mitochondrial dysfunction, and expression of caspase-3 were seen to be significantly increased on MPTP intoxication. Furthermore, the phosphorylation of Akt, ERK1/2, and GSK3*β* was also reduced by the neurotoxic action of MPTP. However, CGA was potent in protecting the neurons against MPTP-induced cytotoxicity through the phosphorylation of GSK3*β* via activation of Akt and ERK1/2 signalling pathways in the mouse model of PD. Thus, this study helps to understand the neuroprotective mechanism of CGA in PD which can be further explored for clinical interventions.

## Data Availability

The data used to support the findings of this study are available from the corresponding author upon request.
